# Gender Dimorphism Does Not Affect Secondary Compound Composition in *Juniperus communis* After Shoot Cutting in Northern Boreal Forests

**DOI:** 10.3389/fpls.2018.01910

**Published:** 2018-12-21

**Authors:** Sari Stark, Françoise Martz

**Affiliations:** ^1^Arctic Centre, University of Lapland, Rovaniemi, Finland; ^2^Production System Unit, Natural Resources Institute Finland (Luke), Rovaniemi, Finland

**Keywords:** *Juniperus communis*, phenolics, terpenoids, compensatory growth, secondary metabolism, boreal forest

## Abstract

Due to a difference in plant resource allocation to reproduction, the males of dioecious plants may be more growth-orientated, whereas females may allocate more resources for synthesizing secondary compounds. This mechanism is considered to cause gender-specific differences in the plant responses to the loss of plant biomass. Here, we tested gender dimorphism in the responses of common juniper (*Juniperus communis*) to shoot cutting in four juniper populations located in northern boreal forests in Finland. We collected shoots from uncut junipers and from junipers subjected to shoot cutting in the previous year, and analyzed them for their shoot growth as well as phenolic and terpenoid concentrations. There were no differences in foliar phenolic or terpenoid concentrations between the males and the females. Shoot cutting increased phenolic but not terpenoid concentrations, similarly, in both males and females. Our study reveals that the nature of gender dimorphism may differ among species and locations, which should be considered in theories on plant gender dimorphism. Given the similar phenolic and terpene concentrations in both genders, the different sexes in the northern juniper populations might experience equal levels of herbivory. This lack of gender dimorphism in biotic interactions could result from the high need of plant secondary metabolites (PSM) against abiotic stresses, which is typical for juniper at high latitudes.

## Introduction

It has long been considered that in dioecious plant species, the growth rates and chemical defenses may differ between female and male individuals (Ågren et al., 1999; Cornelissen and Stiling, 2005; Avila-Sakar and Romanow, 2012). The gender-specific differences in plant carbon allocation likely result from a higher resource investment to reproduction in females, which may lead to a lower growth rate and higher concentrations of plant secondary metabolites (PSM) in females compared with males. Gender dimorphism is also suggested to influence plant responses to the loss of biomass in response to herbivory (Cornelissen and Stiling, 2005). In slow-growing woody plants, the loss of biomass often induces an increase in PSMs, which constitutes an important part of the plant resistance against invertebrate herbivory (Haukioja and Koricheva, 2000) and mammalian browsing (Bryant et al., 1991, 2014). Whether this is a defense reaction or an indirect result of environmental constraints that regulate the trade-off between plant growth and synthesis of secondary phenolic compounds has remained unclear (Tuomi et al., 1990; Jones and Hartley, 1999; Mattson et al., 2005). The capacity of plants to compensate for the lost biomass through increased growth rates (i.e., plant compensatory growth) constitutes another important means of plant tolerance to herbivory (Lehtilä, 2000; Tiffin, 2000; Peinetti et al., 2001; Cromsigt and Kuijper, 2011). Owing to gender dimorphism, males may more commonly respond to the loss of plant biomass by compensatory growth, whereas females respond by increasing plant secondary compounds (Cornelissen and Stiling, 2005).

The secondary compounds in plants exhibit a diverse spectrum of biological functions, and several factors regulate their synthesis (Koricheva et al., 1998; Stamp, 2003; Theis and Lerdau, 2003). Plant secondary metabolites can roughly be gathered in three classes of chemical compounds, namely alkaloids, phenolic compounds and terpenes, with 1000s of compounds in each class (Chomel et al., 2016). PSMs have important roles in plant development, plant–plant and plant–microbe/insect/herbivore interactions; they also govern the mechanisms of allelopathy, influencing intra- and interspecific competition between plants. Despite well-established importance of gender dimorphism, the majority of studies investigating the effects of environmental stresses on PSMs has focused on plant phenolics (e.g., Cornelissen and Stiling, 2005) while less is known about gender dimorphism of terpenoids. For understanding the ecological consequences of gender-specific differences on plant growth and survival, more studies are needed on the role of sexual dimorphism that would consider the multiple functions of the different classes of PSMs.

Common juniper *(Juniperus communis)* of the Cupressaceae family is an evergreen dioecious gymnosperm shrub that has one of the widest global distributions of any gymnosperm (Thomas et al., 2007). In Europe, the distribution of juniper ranges from the Mediterranean to the Arctic and within each area, is found in a very wide range of habitats, such as old pastures, forests, and peatlands. Juniper foliage is rich in secondary substances, particularly terpenoids and phenolics (Martz et al., 2009). Previous studies have demonstrated strong gender dimorphism in juniper. For example, juniper populations may be biased toward males, because females have higher mortality rates in resource-limited conditions (Ortiz et al., 2002). The males of *Juniperus thurifera* start flowering younger (Gauquelin et al., 2002). Different juniper genders also exhibit differing physiological responses to shading from neighboring plants (Verdú et al., 2004). Some studies on junipers, however, have not found gender-specific differences in growth or survival (Marion and Houle, 1996; Ortiz et al., 2002), or these differences have been highly site-specific (DeSoto et al., 2016). Northern boreal juniper populations provide an excellent opportunity to investigate gender dimorphism in plants, because these populations grow in stressful environments and have high concentrations of both phenolics and terpenes in their biomass (Martz et al., 2009).

**FIGURE 1:**
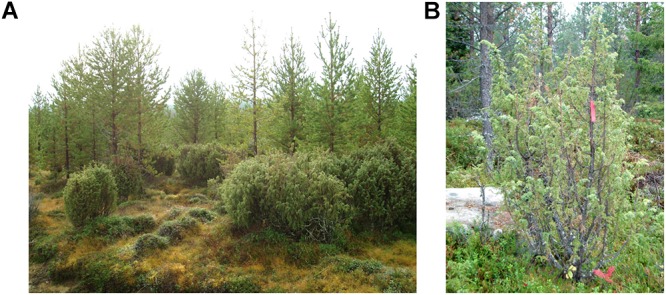
Junipers in northern boreal forest sites (**A**: Savukoski, **B**: Salla) in Finland.

Here, we analyzed gender dimorphism in juniper by analysing the secondary compounds and responses to shoot cutting in northern boreal forests in Finland. In northern boreal forest, junipers commonly form polycormic tall shrubs instead of trees (Figure 1). We assumed that if there are differences in the reproductive effort between the genders, male and female individuals should differ in growth rates and the concentrations of PSM as well as in their responses to a loss of shoot biomass. We predicted that (1) concentrations of secondary metabolites should be higher whereas growth rates lower in females than males. As woody plants respond to biomass loss both by compensatory growth and production of secondary metabolites, we further predicted that (2) shoot cutting should induce a stronger increase in the concentrations of PSMs in females than males, whereas the compensatory growth after biomass loss should be higher in males than females.

## Materials and Methods

### Study Sites and Selecting Junipers for Study

For our investigation, we selected four sites in northern Finland with dense juniper population in a uniform area (Table 1). All sites are located in the northern boreal vegetation zone and can be classified as mid-successional boreal forests with Scots pine (*Pinus sylvestris*) as the dominant tree species. We compared the role of gender and the loss of biomass in these populations, because the local communities had used these sites for shoot collection for natural products. Common juniper extract is used in some pharmaceutical and technical preparations, cosmetic products, and as a food additive (Mäkitalo et al., 2006; Stark et al., 2010). As part of an applied research project to investigate the recovery rate of junipers from shoot gathering and for creating recommendations for sustainable gathering (see Stark et al., 2010), we searched for several sites with a history of commercial shoot gathering during years 2002–2004. In each site, we randomly chose male and female junipers in a collected area and in an adjacent uncollected area (i.e., ranging from a few 100 m to 5 km; Table 1). Individuals with shoot cut in 2004 – generally implemented by cutting with knives or by striping – were identified in collaboration with collectors. In the sites used for shoot gathering, the collectors generally gather shoots from each juniper as they go through the area. Consequently, the different juniper individuals are subjected to shoot cutting in a randomized way that does not depend on juniper characteristics such as size or growth rate. When selecting juniper individuals for our study, we also ensured that each of them was cut by a visual identification of cutting marks.

**Table 1:** Study sites and their geographical location.

			Total number
Site #	Site name	Coordinates	of samples
S1	Rovaniemi	66°30′N, 25°44′E	40
S2	Keminmaa	65°48′N, 24°32′E	24
S3	Salla	66°50′N, 28°40′E	32
S4	Savukoski	67°17′N, 28°09′E	32


*The total number of samples includes all individuals (males and females in the uncut and cut areas within each site).*

### Sampling

The chemical quality of needles and shoot growth rates were collected and analyzed in July 2005, 1 year after the previous shoot cutting. In the field, morphological data were recorded on the selected junipers [*N* = 6 per treatment in Keminmaa (S1), *N* = 10 in Rovaniemi (S2), *N* = 8 in Salla (S3), and Savukoski (S4)]. For each shrub, we recorded: (1) height of tree, (2) diameter of the crown of the shrub from two directions, which, together with height, was later used for calculating an approximation of juniper shrub volume, (3) dry weight of the current year shoots, (4) needle coverage (%), (5) percentage of top dead shoots. We collected a sample of 15–40 (depending on the size of the juniper) current-year shoots from each individual. Current-year shoots (including stem) were stored in paper bags and transported to the laboratory within 1–2 days where they were immediately air dried (60°C 1 day), milled and stored in sealed plastic bag at +4°C in the dark until analysis. We analyzed the mean fresh and dry weight (drying at 60°C for 48 h) of the shoot samples, indicative of their growth rate, by calculating the number of shoots and analysing the total weight of the dried samples.

### Chemical Analyses

PSM were extracted from dry powder and analyzed as previously described (Martz et al., 2009). Briefly a one-step extraction protocol was developed to extract terpenoid in hexane and soluble phenolics in methanol 75%. Milled juniper needles (0.5 g) were extracted with 4 ml of methanol:H_2_O (3:1, v/v) + 4 ml n-hexane by shaking 2 h in the dark at room temperature. Isoborneol (200 μg) was added before shaking as internal standard for terpenoids.

After centrifugation, the upper organic fraction containing terpenoids was removed, concentrated and analyzed by gas chromatography with a HP-5 (30 m × 320 μm × 0.25 μm) column (Agilent Technologies, Santa Clara, CA, United States) using the following conditions: injector 200°C, flame ionization detector 280°C, helium as carrier gas (1 ml/min) and the following temperature programme: 50–80°C at 15°C/min, 80–100°C at 3°C/min, 100–160°C at 10°C/min, 160–200°C at 3°C/min, 200–250°C at 15°C/min. Terpenoids were identified and quantified as described previously (Martz et al., 2009). The internal standard isoborneol was used for calculation of the efficiency of recovery of each sample during the whole extraction process. Limonene and α-pinene were used to draw calibration curves for all monoterpenes (average curve used) and β-caryophyllene was used, similarly, for all sesquiterpenoids. Only monoterpenoids and sesquiterpenoids were quantified in this study, and their sum labeled as “terpenoid content.”

Soluble phenolics present in the lower aqueous fraction were analyzed by HPLC (Waters, Milford, MA, United States) with a Spherisorb ODS II column (4.6 × 250 mm, particle size 5 μm) column (Waters, Milford, MA, United States) using a binary solvent system (solvent A: 1% ammonium formiate, 10% formic acid in water; solvent B: 1% ammonium formiate, 10% formic acid in methanol). The elution programme was as follow: 0–5 min: 0% B, 5–45 min: 0–100% B, 45–86 min: 100% B; 86–90 min: 100–0% B, 90–120 min: 0% B, at 35°C and at 1 ml/min. Detection and quantification were made at 280 nm using a UV/visible diode-array detector (Waters PDA 996). The following compounds were used to draw calibration curves for quantification: catechin (for proanthocyanidins and unknown compounds), rutin (for all flavonols) and apigetrin (for all flavones).

### Calculation and Statistics

**FIGURE 2:**
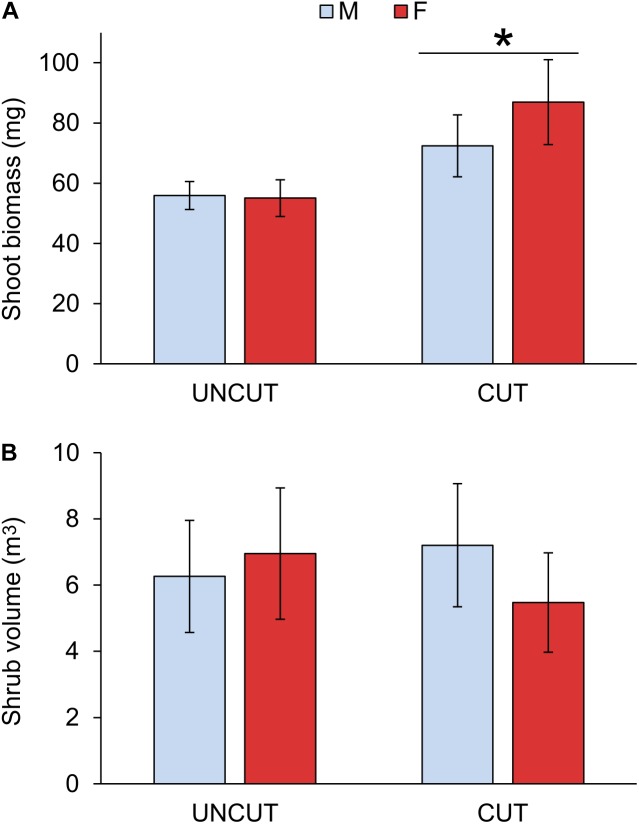
Effect of cutting and gender on shoot biomass **(A)** and volume of the shrub **(B)**. UNCUT = uncut junipers, CUT = junipers subjected to shoot cutting in the previous year. Values are mean ± S.E. (*n* = 4 sites). A star indicates statistically significant cutting effect at *p* = 0.001.

The volume of the individual juniper plants was computed using the height and the averaged width (2 values). The raw data have been used in Figures 1, 2; values are the mean of the four sites ± SE of the mean. For statistical analysis, data were transformed when required to meet the assumption of normality. Log10 transformation was applied to shoot biomass, volume of the shrub, total phenolics, % of monoterpenes, Grp3, Grp4, Grp5, U1, α-pinene. Square root transformation was used for total terpenoids and the % of U2. The experiment followed a block-design, with site used as blocks. The Linear Mixed Model was used with gender, cutting and their interaction as fixed factors and site as a random factor. Variance component was used as a covariance structure. All statistical testing was conducted with the IBM SPSS Statistics Software (Version 25.0, SPSS Inc., Chicago, IL, United States).

## Results

### Composition of Phenolics and Terpenoids in the Common Juniper Shoots

The chemical composition of juniper shoots was similar to that previously reported from samples in Finland (Martz et al., 2009). Thirty-six peaks were identified by HPLC analysis. According to their UV spectra and comparison with authentic standards, the peaks were gathered into seven groups. In order of decreasing abundance, we identified: proanthocyanidins (PAs) (including catechin; Group 1), flavones (mainly apigenin derivatives; Group 2), flavonols (including quercetin derivatives; Group 3), and unidentified compounds (“others,” Group 4). Although unidentified as well, a few compounds with similar spectra were detected and thus were not included in group 4: group 5 (two compounds with λmax of 284 and 311 nm) and group 6 (two compounds with λmax of 280, 296, and 305 or 277, 305, and 328 nm). In addition, two unidentified individual compounds, which were not included in group 4 because of their significant abundance, were detected as follows: U1 (λmax = 266 nm with a shoulder at 300 nm, possibly a neolignan) and U2 (λmax = 273 nm) (Martz et al., 2009; Table 2 and Supplementary Table S1). The terpenoid composition appeared much more variable and the most abundant compounds were of germacrene types (B, D-ol, D) (sesquiterpenoids), α-pinene and several unknown compounds.

**Table 2:** Phenolic and terpenoid composition in juniper shoots and statistical significance of cutting, gender, or cutting by gender interaction effects calculated using the Mixed Linear Model.

Compounds	Concentration (%)				*F*-value		
	Uncut		Cut		Cutting	Gender	Cutting ^∗^gender
	M	F	M	F			
**Phenolics**							
PA (1)	22.7 ± 1.1	23.2 ± 1.3	22.6 ± 1.4	23.2 ± 1.4	0.035	0.694	0.008
Flavones (2)	16.7 ± 0.7	16.0 ± 0.7	17.3 ± 0.9	17.1 ± 0.9	2.306	0.753	0.272
Flavonols (3)	17.9 ± 0.8	17.9 ± 0.8	19.7 ± 1.0	18.9 ± 1.0	5.988↑	0.365	1.221
Others (4)	20.9 ± 0.6	20.2 ± 0.7	19.5 ± 0.7	19.5 ± 0.6	8.283^∗∗^↓	1.181	1.981
Group 5	8.0 ± 0.3	8.0 ± 0.4	7.0 ± 0.3	6.9 ± 0.3	17.824^∗∗^↓	0.072	0.053
Group 6	4.7 ± 0.2	4.9 ± 0.3	5.3 ± 0.3	5.3 ± 0.6	9.286↑	0.571	0.309
U1	4.4 ± 0.7	5.3 ± 0.8	4.3 ± 0.6	4.5 ± 0.6	1.760	0.022	1.101
U2	4.7 ± 0.3	4.4 ± 0.2	4.4 ± 0.4	4.6 ± 0.4	1.056	1.142	0.307

**Terpenoids**							
Monoterpenes	17.5 ± 2.1	15.0 ± 1.7	13.5 ± 1.8	13.4 ± 1.3	1.619	0.205	0.627
Sesquiterpenes	82.5 ± 2.1	85.0 ± 1.7	86.5 ± 1.8	86.6 ± 1.3	–	–	–
Germacrene	31.9 ± 2.1	36.8 ± 2.1	30.6 ± 1.8	32.4 ± 1.4	3.256	4.227↑	0.823
α-pinene	10.3 ± 1.6	8.3 ± 1.3	7.8 ± 1.3	7.7 ± 1.0	0.354	0.509	0.748


*Concentrations are expressed as % of total soluble phenolic or terpenoid content, respectively (values are mean ± SE, n = 31). Statistical significant effects are indicated by ^∗∗^ (p < 0.01) or ^∗^ (p < 0.05). An arrow indicates the direction of the change: CUT compared to UNCUT, and FEMALE compared to MALE.*

### Effect of Cutting on Growth Rate

A significant increase in shoot biomass of juniper shoots was measured due to previous-year cutting (Figure 2 and Table 3). In the uncut plots, male and female did not show any difference in growth. However, females tended to have a higher growth rate after cutting, although the interaction between cutting and gender was only marginally significant (*P* = 0.085; Table 3 and Supplementary Table S2). Previous-year cutting did not affect the needle coverage nor the proportion of top dead shoots (Table 3). The site had no significant effect on modeling the shoot biomass, volume of the shrub, needle coverage or top dead shoots (Wald *Z*-test: *p* = 0.241, 0.256, 0.468, and 0.857, respectively).

**Table 3:** Statistical significance of cutting, gender and their interaction on growth and secondary compounds in juniper shoots calculated using the Linear Mixed Model (see Materials and Methods).

Variable	Fixed effect	*F*-value	*p*-value
**Growth**			
Shoot biomass	Cutting	30.464	**<0.001^∗∗^**
	Gender	0.551	0.460
	Cutting^∗^gender	3.015	0.085
Volume	Cutting	0.270	0.605
	Gender	0.192	0.662
	Cutting^∗^gender	0.849	0.359
Needle coverage (%)	Cutting	0.009	0.923
	Gender	1.242	0.268
	Cutting^∗^gender	0.019	0.891
Top dead shoots (%)	Cutting	0.002	0.961
	Gender	0.010	0.921
	Cutting^∗^gender	0.104	0.748

**Secondary Compounds**			
Total phenolics	Cutting	14.838	**<0.001^∗∗^**
	Gender	0.849	0.359
	Cutting^∗^gender	0.029	0.865
Terpenoids	Cutting	2.492	0.117
	Gender	0.653	0.421
	Cutting^∗^gender	0.937	0.335


*Statistical significant effects are indicated by ^∗∗^ (*p* < 0.01) or ^∗^ (*p* < 0.05).*

### Effect of Cutting on PSM

**FIGURE 3:**
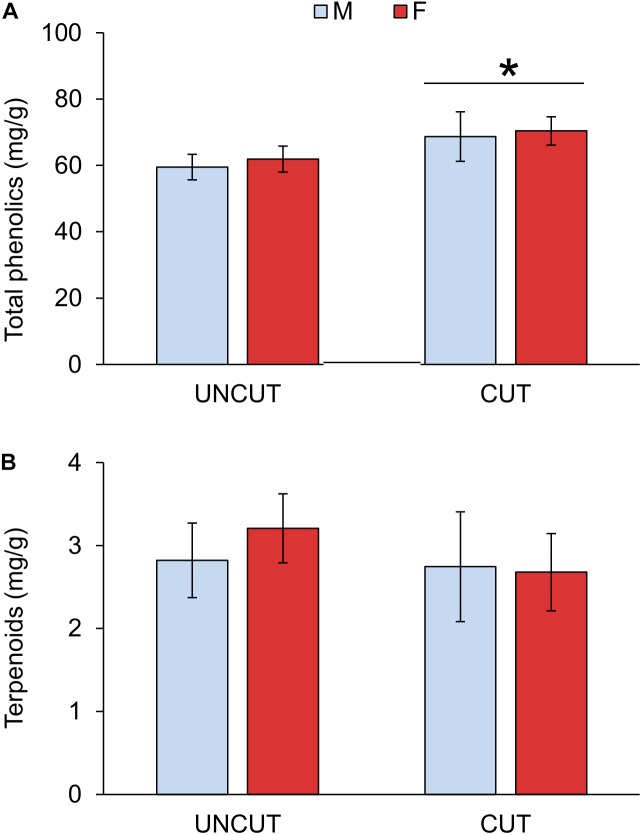
Effect of cutting and gender on total soluble phenolics **(A)** and terpenoids **(B)** concentrations in juniper shoots. UNCUT = uncut junipers, CUT = junipers subjected to shoot cutting in the previous year. Values are mean ± S.E. (*n* = 4 sites). A star indicates statistically significant cutting effect at *p* = 0.01.

Previous-year cutting significantly increased the total phenolic content in juniper shoots in both male and female individuals (Figure 3 and Table 3 and Supplementary Table S2). The phenolic composition was as well affected by cutting with significant increases in flavonols and compounds of Group 6 as well as decreases in abundances of compounds in Groups 4 and 5 (Table 2). Flavonols represent a major group of phenolics in juniper shoots, and a detailed analysis of the individual compounds showed that 3 compounds of the four flavonols detected increased in response to cutting (Supplementary Table S1). Only hyperin, the most abundant flavonol, was not affected by cutting. Although the abundance of all flavones (Group 2) did not significantly increase in response to cutting, two specific compounds (apigenin derivative 1: λmax 272, 332 nm and U31: λmax 268, 335) were more abundant in shoots from collected junipers. Several compounds in Group 4 showed the same decreasing abundance after cutting. Two other unknown compounds (U11 in Group 5: λmax 291, 316 nm and U27 in Group 6: λmax 277, 305, 328 nm) were as well significantly affected due to cutting (Supplementary Table S1). Neither the terpenoid content nor its composition was affected by cutting (Tables 2, 3). The site had no significant effect on modeling the phenolic or terpenoid concentrations (Wald *Z*-test: *p* = 0.252 and 0.241, respectively).

### Effect of Gender Dimorphism on PSMs

Generally only little significant difference was observed between males and females in uncut areas and response to cutting (Tables 2, 3 and Supplementary Table S2). Only one phenolic (U18: λmax 280, 367 nm, in Group 4) was marginally more abundant in males than females shoots (Supplementary Table S1). A gender-specific difference was observed in the abundance of germacrenes with significantly higher abundance in females compared to males, especially in uncut areas (Table 3).

## Discussion

Studies on dioecious plants have documented gender-related differences in growth rates and concentrations of secondary metabolites with higher growth but lower PSM concentrations in males compared with females (e.g., Ågren et al., 1999; Cornelissen and Stiling, 2005). Consequently, in dioecious plant species, the intensity of herbivory may be biased toward males (Cornelissen and Stiling, 2005). Studies have found exceptions to these generalizations (Avila-Sakar and Romanow, 2012), and among the species belonging to the dioecious *Juniperus* genus, previous evidence on gender dimorphism has also been mixed. Contrasting the generalization, Massei et al. (2006) found higher concentrations of terpenes and phenolics in male than female individuals in Mediterranean *J. oxycedrus macrocarpa*, and McGowan et al. (2004) that the females of prostrate juniper *J. communis* ssp. *nana* in northern Scotland were more frequently subjected to herbivory than males. No gender-specific difference in juniper growth was observed along an altitudinal gradient in Sierra Nevada despite decreasing reproductive success along this gradient (Ortiz et al., 2002). Here, except for a few individual compounds, we found no effects of gender in phenolic and terpenoid concentrations in *J. communis* in northern boreal forests. Although, as predicted, shoot cutting significantly increased phenolic concentrations, the level of PSMs increased, similarly, in both males and females. Further, theories on gender dimorphism state that males should be more growth-orientated than females (e.g., Ågren et al., 1999; Cornelissen and Stiling, 2005), but we found marginally (*P* < 0.10) higher growth after shoot cutting in females compared with males.

The lack of clear gender dimorphism in PSMs and growth in northern juniper populations compared with e.g., boreal deciduous trees (Nissinen et al., 2018; Ruuhola et al., 2018; Zhang et al., 2018) is noteworthy, because junipers in these systems seem to have a particularly high level of chemical defense. Along a large geographical gradient from the southern to northern boreal forests, Martz et al. (2009) found that secondary compound concentrations in the common juniper significantly increased with latitude. Interestingly, these trends were similar in both terpenoids and phenolics, although these compound groups exert largely differing ecological functions. Terpenoids may have a key role in the defense against mammalian herbivory (Mutikainen et al., 2000), and the low palatability of juniper to herbivores is commonly derived from oils found in the needles, cones and wood, dominated by monoterpenes (Thomas et al., 2007). Despite the low palatability, junipers are commonly subjected to herbivory in different habitats (Livingston, 1972; Miller et al., 1982; Fuhlendorf et al., 1997), and in the boreal forests, herbivory on juniper is dominated by shoot consumption by moose (*Alces alces*) during the winter. Contrasting with terpenoids, phenolics may have a higher importance in the plant protection against invertebrate herbivory (Koricheva et al., 1998), and photooxidative stress (Close and McArthur, 2002). The need for antioxidative compounds in relation to light intensity may increase in conditions of both nutrient deficiency and low temperature (Close and McArthur, 2002), which could explain why their concentrations increase with latitude (Stark et al., 2008; Martz et al., 2009, 2010). Traditional theories also suggested that greater concentrations of phenolics may result from allocation of extra photosynthesized carbon to secondary metabolites as a result of nutrient deficiency (Bryant et al., 1983; Mattson et al., 2005).

Although both phenolic and terpenoid concentrations in juniper seem to show a similar gradient with increasing latitude (Martz et al., 2009), here, we found that the responses of PSM concentrations to shoot cutting varied greatly among the different compounds and compound groups. More specifically, cutting lead to increased concentrations of soluble phenolics and a higher abundance of flavonoids (specific flavones and flavonol glycosides). As we analyzed samples 1 year after the shoot cutting, our analyses depict delayed inducible reactions that take place after a time lag from the loss of biomass (Tuomi et al., 1990). Although previous studies have indicated that delayed inducible reactions in conifers is relatively uncommon when compared with the deciduous trees (Nykänen and Koricheva, 2004), increasing phenolic concentrations in response to shoot cutting agree with previous findings on e.g., *Pinus* species (Honkanen et al., 1999; Roitto et al., 2008). Flavonols in juniper are mainly quercetin derivatives that exert strong antioxidant activity due to their chemical features (Rice-Evans et al., 1997); thus, shoot cutting led to higher abundances of compounds with a high antioxidant capacities that are more efficient in relation to the carbon cost to their synthesis (*sensu* Close and McArthur, 2002).

Contrasting with phenolics, we found no overall shoot cutting effect on terpenoids. In line with our investigation, the previous study by Honkanen et al. (1999) also found increasing phenolic concentrations in response to defoliation in boreal coniferous tree *Pinus sylvestris* L. but no effects on terpenoids, despite they are considered the main class of anti-herbivore defensive compounds. Contrasting with this idea, a meta-analysis by Nykänen and Koricheva (2004) concluded that the damage in woody plants quite commonly reduces the concentrations of terpenes. This may be true also in the case of juniper, as reduced yields of essential oils in junipers after a severe browsing damage have previously been found (Markó et al., 2008). Condensed tannins and flavonoids, among many other phenolic compounds are derived from phenylalanine via the phenylpropanoid pathway (Vogt, 2011) and terpenoids are synthesized via acetyl-CoA, pyruvate and glyceraldehyde-3-phosphate in parallel cytosolic of plastid metabolic pathways (Singh and Sharma, 2015). This shows that disturbance of the general carbon metabolism due to, for example biotic/abiotic stress or compensatory growth will have consequences on the PSM content.

Although the mechanisms underlying our findings remain uncertain, our study adds to previous evidence showing unclear or ambiguous effects of gender on growth, secondary compound concentrations and reproduction in *Juniperus* species (e.g., Marion and Houle, 1996; Ortiz et al., 2002; Verdú et al., 2004; Massei et al., 2006; DeSoto et al., 2016). For example, aged juniper populations are male-biased, but this bias does not seem to be easily explained by the gender-specific differences in the cost of reproduction (Gauquelin et al., 2002; Ortiz et al., 2002). Earlier reviews have already concluded that there is a need to revise theories predicting how gender dimorphism affects plant performance and responses to environmental stresses (Avila-Sakar and Romanow, 2012; Vega-Frutis et al., 2013). For woody plants, the tolerance of herbivory is a major component of plant resistance, because the probability of herbivory is high due to large size and long life span. However, the recovery potential of these species could be driven by the type of herbivory they commonly experience (Haukioja and Koricheva, 2000). Noteworthy, García et al. (2001) found that frugivory at *Juniperus communis* in the Mediterranean mountains depended largely on population characteristics rather than on individual attributes. If herbivory, such as browsing by moose, is commonly centered on locations with numerous and dense juniper populations to provide large food quantity, the chemical quality of the different individuals might not exert a primary role in the food selection of herbivores. Under these conditions, the capacity for regrowth could outweigh the importance of PSMs in herbivory tolerance. Further, the lack of clear gender dimorphism in northern boreal juniper populations could also result from the high need of PSMs at high latitude to protect from abiotic stresses (Martz et al., 2009). Although herbivory might even constitute one of the driving forces behind the evolution of dioecy in plants (Bawa, 1980; Cornelissen and Stiling, 2005; Avila-Sakar and Romanow, 2012; Vega-Frutis et al., 2013), studies on juniper populations have demonstrated that gender-related differences in growth and resource storage may be a consequence of local adaptation to environmental conditions (DeSoto et al., 2016). Protection against abiotic stresses through the PSMs could have such major significance for plant success under northern conditions that it might override any gender-specific differences in the carbon allocation for synthesizing PSMs.

Ecological factors that limit plant success in each specific conditions could also explain why we detected a marginally higher capacity for compensatory growth in females despite a presumably higher resource cost for reproduction (*sensu* Cornelissen and Stiling, 2005). Over a large geographical gradient from the Mediterranean to the sub-Arctic vegetation zone across Europe, García et al. (2000) concluded that the juniper population viability in the north may in fact be under less pressure compared with juniper populations at mid-latitudes, because these populations are free from seed predation. Experimental evidence on dioecious plants has suggested that increasing resources may weaken gender-specific differences within plant species, and consequently, the gender-specific differences in phenolic concentrations could be pronounced under high resource limitation (Palumbo et al., 2007). Further, woody plants seem to recover from the loss of biomass better under low than high resource availability, possibly because under these conditions, plants generally grow below their potential maximum growth rate (Hawkes and Sullivan, 2001). As northern conditions with low nutrient availability, low temperatures and high light likely require high defense through PSMs (Martz et al., 2009), the combination of high need for chemical defense and low pressure on reproduction (García et al., 2000) might direct gender dimorphism from gender-specific differences in defense toward the importance of compensation after the loss of biomass.

## Author Contributions

SS designed the experiments and performed field sampling and measurements. FM ran the laboratory analyses and conducted statistical tests. SS and FM jointly wrote the manuscript.

## Conflict of Interest Statement

The authors declare that the research was conducted in the absence of any commercial or financial relationships that could be construed as a potential conflict of interest. The handling Editor declared a shared affiliation, though no other collaboration, with one of the authors FM at the time of review.
