# Supplementary material for: Gender Dimorphism Does Not Affect Secondary Compound Composition in Juniperus communis After Shoot Cutting in Northern Boreal Forests
Source: Front Plant Sci. 2018 Dec 21;9:1910. doi: 10.3389/fpls.2018.01910 (PMC6308805; doi:10.3389/fpls.2018.01910)
Supplement: Supplementary file 1 [file Table_1.DOCX]

**Table S1:** Individual phenolic concentrations in juniper shoots and statistical significance of cutting, gender, or cutting by gender interaction effects calculated using the Mixed Linear Model. Concentrations are expressed as % of total soluble phenolic (n=31, value = mean ± SE). Statistical significant effects are indicated by ** (p<0.01) or * (p<0.05). An arrow indicates the direction of the change. Compounds are listed in their decreasing abundance.

| **Compounds** | **Concentration** | | | | | | | | | | **F-value** | | |
| --- | --- | --- | --- | --- | --- | --- | --- | --- | --- | --- | --- | --- | --- |
|  | Uncut | | | | | Cut | | | | | Cutting | Gender | Cutting  * gender |
|  | M | | F | | | M | | | F | |  |  |  |
| **PA (Group 1)** | | | | | | | | | | | | | |
| CAT | 9.18 ± | 0.61 | | 9.51 ± | 0.75 | | 9.29 ± | 0.74 | 9.58 ± | 0.77 | 0.006 | 0.348 | 0.006 |
| U8 | 3.15 ± | 0.19 | | 3.31 ± | 0.19 | | 3.24 ± | 0.23 | 3.21 ± | 0.21 | 1.297 | 0.347 | 0.246 |
| U12 | 2.61 ± | 0.10 | | 2.77 ± | 0.11 | | 2.60 ± | 0.12 | 2.66 ± | 0.12 | 0.766 | 1.463 | 0.193 |
| U7 | 2.52 ± | 0.20 | | 2.62 ± | 0.20 | | 2.52 ± | 0.21 | 2.54 ± | 0.20 | 1.647 | 0.276 | 0.019 |
| B3 | 2.22 ± | 0.09 | | 2.30 ± | 0.10 | | 2.16 ± | 0.09 | 2.37 ± | 0.12 | 0.033 | 3.240 | 0.922 |
| U10 | 1.93 ± | 0.08 | | 1.99 ± | 0.07 | | 1.91 ± | 0.07 | 1.95 ± | 0.08 | 0.694 | 0.679 | 0.109 |
| U5 | 0.46 ± | 0.05 | | 0.39 ± | 0.03 | | 0.34 ± | 0.04 | 0.38 ± | 0.04 | 7.400****↓** | 0.003 | 2.844 |
| U6 | 0.41 ± | 0.02 | | 0.36 ± | 0.02 | | 0.49 ± | 0.09 | 0.50 ± | 0.06 | 3.661 | 0.356 | 2.976 |
| **Flavones (Group 2)** | | | | | | | | | | | | | |
| API_2 | 8.17 ± | 0.34 | | 7.60 ± | 0.35 | | 7.95 ± | 0.42 | 7.87 ± | 0.44 | 0.052 | 0.997 | 0.548 |
| API_1 | 3.58 ± | 0.22 | | 3.45 ± | 0.22 | | 3.93 ± | 0.20 | 3.92 ± | 0.23 | 9.870****↑** | 0.192 | 0.020 |
| U30 | 3.21 ± | 0.15 | | 3.09 ± | 0.13 | | 3.19 ± | 0.20 | 3.18 ± | 0.17 | 0.173 | 0.187 | 0.106 |
| U29 | 1.21 ± | 0.06 | | 1.14 ± | 0.06 | | 1.20 ± | 0.06 | 1.21 ± | 0.07 | 0.844 | 0.537 | 0.761 |
| U31 | 0.76 ± | 0.07 | | 0.77 ± | 0.06 | | 1.00 ± | 0.10 | 0.91 ± | 0.09 | 4.010***↑** | 0.039 | 0.267 |
| **Flavonols (Group 3)** | | | | | | | | | | | | | |
| Hyperin | 10.43 ± | 0.43 | | 10.3 ±4 | 0.36 | | 10.59 ± | 0.58 | 10.20 ± | 0.55 | 0.217 | 0.203 | 0.392 |
| U26 | 4.26 ± | 0.32 | | 4.37 ± | 0.30 | | 5.35 ± | 0.32 | 5.04 ± | 0.36 | 22.005****↑** | 0.229 | 1.598 |
| U23 | 2.00 ± | 0.11 | | 2.00 ± | 0.13 | | 2.45 ± | 0.14 | 2.32 ± | 0.15 | 21.863****↑** | 1.078 | 0.300 |
| U25 | 1.07 ± | 0.07 | | 1.24 ± | 0.21 | | 1.34 ± | 0.12 | 1.35 ± | 0.11 | 9.422****↑** | 0.001 | 0.012 |
| **Others (Group 4)** | | | | | | | | | | | | | |
| U22 | 3.02 ± | 0.11 | | 2.81 ± | 0.14 | | 2.67 ± | 0.11 | 2.71 ± | 0.12 | 7.477****↓** | 1.759 | 3.029 |
| U18 | 2.58 ± | 0.08 | | 2.38 ± | 0.09 | | 2.47 ± | 0.08 | 2.37 ± | 0.06 | 0.498 | 6.148***↓** | 1.037 |
| U14 | 2.36 ± | 0.06 | | 2.28 ± | 0.06 | | 2.36 ± | 0.09 | 2.40 ± | 0.10 | 0.259 | 0.084 | 0.627 |
| U28 | 2.28 ± | 0.12 | | 2.20 ± | 0.09 | | 2.20 ± | 0.17 | 2.00 ± | 0.11 | 5.226****↓** | 0.675 | 0.087 |
| U21 | 2.07 ± | 0.17 | | 2.07 ± | 0.16 | | 1.42 ± | 0.12 | 1.49 ± | 0.12 | 17.452****↓** | 0.170 | 0.147 |
| U16 | 1.83 ± | 0.08 | | 1.79 ± | 0.08 | | 1.67 ± | 0.09 | 1.80 ± | 0.07 | 1.385 | 1.167 | 2.224 |
| U17 | 1.53 ± | 0.11 | | 1.29 ± | 0.11 | | 1.37 ± | 0.08 | 1.31 ± | 0.07 | 0.010 | 5.091 | 3.219 |
| U15 | 1.05 ± | 0.05 | | 1.03 ± | 0.07 | | 1.02 ± | 0.03 | 1.04 ± | 0.05 | 0.196 | 0.114 | 0.577 |
| U20 | 1.61 ± | 0.13 | | 1.75 ± | 0.15 | | 1.71 ± | 0.10 | 1.65 ± | 0.13 | 0.483 | 0.005 | 1.226 |
| U19 | 1.08 ± | 0.07 | | 1.04 ± | 0.06 | | 1.27 ± | 0.10 | 1.20 ± | 0.06 | 11.913****↑** | 0.443 | 0.001 |
| U32 | 0.92 ± | 0.09 | | 0.68 ± | 0.07 | | 0.72 ± | 0.07 | 0.84 ± | 0.07 | 0.122 | 0.266 | 6.128* |
| U3 | 0.43 ± | 0.03 | | 0.44 ± | 0.03 | | 0.35 ± | 0.03 | 0.37 ± | 0.02 | 15.003****↓** | 0.105 | 0.699 |
| U1 | 0.32 ± | 0.09 | | 0.41 ± | 0.13 | | 0.23 ± | 0.04 | 0.33 ± | 0.08 | 0.360 | 0.701 | 1.338 |
| **Group 5** | | | | | | | | | | | | | |
| U11 | 4.94 ± | 0.31 | | 5.09 ± | 0.35 | | 4.01 ± | 0.23 | 3.96 ± | 0.24 | 19.954****↓** | 0.009 | 0.161 |
| U9 | 2.99 ± | 0.15 | | 2.94 ± | 0.17 | | 2.98 ± | 0.19 | 2.95 ± | 0.19 | 0.690 | 0.199 | 0.161 |
| **Group 6** | | | | | | | | | | | | | |
| U27 | 2.67 ± | 0.23 | | 2.83 ± | 0.24 | | 3.26 ± | 0.21 | 3.13 ± | 0.23 | 10.881****↑** | 0.006 | 0.996 |
| U24 | 1.94 ± | 0.09 | | 2.03 ± | 0.11 | | 2.04 ± | 0.11 | 2.21 ± | 0.12 | 1.827 | 1.790 | 0.407 |
