# Supplementary material for: Gender Dimorphism Does Not Affect Secondary Compound Composition in Juniperus communis After Shoot Cutting in Northern Boreal Forests
Source: Front Plant Sci. 2018 Dec 21;9:1910. doi: 10.3389/fpls.2018.01910 (PMC6308805; doi:10.3389/fpls.2018.01910)
Supplement: Supplementary file 2 [file Table_2.DOCX]

**Table S2**: Estimates of main effects CUTTING and GENDER on variables characterizing juniper growth and secondary compounds according to the Linear mixed model. For the cutting effect, CUT was used as a baseline (estimate = CUT versus UNCUT), and MALE was used as a baseline for the gender effect (estimate = FEMALE versus MALE). Transformed data have been used for shoot biomass, volume of the shrub, total phenolics (LOG_10_ transformation) and terpenoids (square root transformation). Standard error, degree of freedom (df), t-value and its significance are also reported.

| **Variable** | **Parameter** | **Estimate** | **SE** | **df** | **t** | **sig.** |
| --- | --- | --- | --- | --- | --- | --- |
|  |  |  |  |  |  |  |
| Shoot biomass (LOG) | Intercept  Cutting  Gender | 1.89  0.19  0.06 | 0.06  0.04  0.04 | 3.88  117.03  117.03 | 29.514  -5.174  -1.768 | <0.001  <0.001  0.080 |
| Volume (LOG) | Intercept  Cutting  Gender | 6.53  -0.10  -0.10 | 0.14  0.10  0.10 | 4.74  120.07  120.07 | 46.919  1.023  0.966 | <0.001  0.308  0.336 |
| Needle coverage | Intercept  Cutting  Gender | 70.61  -0.58  -3.08 | 3.12  3.44  3.44 | 6.75  96.07  96.07 | 22.615  0.168  0.895 | <0.001  0.867  0.373 |
| Top dead shoots | Intercept  Cutting  Gender | 18.82  -0.96  -0.58 | 2.62  3.61  3.61 | 19.91  95.04  95.04 | 7.178  0.267  0.160 | <0.001  0.790  0.873 |
| Total phenolics (LOG) | Intercept  Cutting  Gender | 1.84  0.06  0.01 | 0.03  0.02  0.02 | 4.62  120.04  120.04 | 56.217  -2.614  -0.533 | <0.001  0.010  0.595 |
| Terpenoids (SQRT) | Intercept  Cutting  Gender | 1.57  -0.17  -0.01 | 0.17  0.09  0.09 | 3.79  120.01  120.01 | 9.144  1.808  0.113 | <0.001  0.073  0.910 |
